# German Review

**Published:** 1891-11

**Authors:** Leonard Pearson


### SELECTIONS FROM FOREIGN JOURNALS.

### *GERMAN AND FRENCH.*

By LEONARD PEARSON, B. S., V. M. D.

### EXPERIMENTS WITH THE MAXIMILIAN METHOD OF TREATING COMPLETE TOE AND QUARTER CRACKS.

Army Veterinarian—Raid.

[*Zeitschrift für Veterinärkunde, Vol. iii., No. 6.*]

During the manœuvres of last summer a cavalry horse, with contracted heels, developed internal quarter cracks on each fore hoof. The following treatment was employed: The shoes were removed, the edge of the wall leveled and the wall thinned, from the coronet to the sole, for a distance of 4 c.m. on each side of the crack. The edges of the crack were smoothed and the sensitive tissues under it were freed from horn. The horse was then shod with bar shoes, the exposed laminæ coated with pine tar and a pressure bandage applied. The hoof was kept moist and the bandage renewed daily, for three days. After four days the bandage was no longer necessary, because the exposed soft parts had become covered with a thin layer of horn. In five days the horse could be exercised and in eight days was ready for service. During the manœuvres the horse was used daily and the quarter cracks healed completely.

Another horse, with regularly shaped but somewhat spreading hoofs, acquired quarter cracks on both front feet, some six days before the manœuvres. In spite of the fact that the crack bled, at first, and caused great pain the horse recovered under this treatment, although it was given hard work every day.

Since July, 1890, I have treated 27 cracks of the hoof, by this method and have been able to cure every case. After having once healed none of them have re-appeared.

The kind of hoofs, upon which the cracks developed, were the following :

1. Regularly shaped hoofs, 8 times.
2. Flat hoofs, 9 times.
3. Hoofs contracted at the heels, 6 times.
4. Short (stumpy) hoof, 1 time.
5. Medium, or half broad half narrow, 3 times.

After the operation, the soft parts soon became covered with horn and the cracks filled out gradually.

An excess of horn always forms on the coronet, which must be removed, with a rasp, every third day. If the crack was very near the heel, the wall would be cut down so that it would not press on the shoe ; while if the crack was on the toe, or side, a crescent-shaped piece would be cut out of the wall, in order to remove press[illegible]om

It is interesting to note that some of these cracks were of six or more years duration and had been treated with all sorts of devices and methods without success, but that they were healed at once by this operation.

In some of these cases the horn had become bent in at borders of the cracks. All of the 27 hoofs are now of a better shape than they were, the heels are broader and the gait of the horses is freer.

I allow the bar shoes to remain from five to six months. From my experience I have no hesitation in recommending the operation in the highest terms.

[This method for the treatment of toe cracks and quarter cracks has been taught and employed at the Veterinary Department of the University of Pennsylvania, for the past six years, and has given the best of results. Translator.]

---

### Erysipelas in a Cow.
By Lucet.

[*Recueil de mèdecine Vétérinaire, Vol. viii, No. 7.*]

This disease, which is exceedingly rare in cattle, commenced in the neighborhood of a tumor, about the size of a hen's egg, on the cows head.

The inflammation and œdema spread rapidly over the head, neck, breast and limb. The temperature reached 41.2° C. Internal and external treatment was of no avail and the animal died, after a sickness of fourteen days.

Microscopical examinations were made of the contents of the swellings and it was found to contain, among other micro-organisms, streptococci, which resembled the organisms of erysipelas of man. (*Berliner Thierarz. Woch.*, *vii.*, *34.*)

---

ACTION OF GLYCERIN IN THE RECTUM.

By Fortuna.

[*Progrès vét. 1890*, *9.*]

A number of authors have recommended clysters of glycerin, in doses of 5 grms. for the horse and ½ grms. for the dog, as an excellent evacuant. As some did not obtain as good results as others, Fortuna conducted a series of experiments on 40 horses, 3 cows, 4 calves, and 14 dogs with the object of determining its real value. In some of the animals the experiments were repeated ten times. The results were as follows: In healthy animals an action of the bowels could be produced in from ½ to 35 minutes (rarely as long as one hour), by which hard (never soft) excrement would be voided. Fortuna was able to show that even this action did not take place unless the rectum was full. In cases of constipation of the horse this remedy was not sufficient to produce an action, but in the dog, sometimes, caused the evacuation of the contents of the rectum. The experiments lead the experimentor to conclude that glycerin, even in doses as large as 15 gm., has no purgative action in the horse. Its action is a simple local irritation of the mucous membrane of the rectum. (*Berl. Thie. Woch.*, *vii.*, *38.*)

---

DISTURBED LOCOMOTION (ATAXIA) IN A HORSE.

By Magnin.

[*Recueil de Méd. Vét.*, *Vol. vii.*, *No 11.*]

A Hungarian saddle horse, which was very susceptible to attacks of colic, was brought to the writer for treatment for this disease.

The rider experienced difficulty in keeping his seat, as the animal kicked continuously. In the stable, the horse stood with the back arched and the hind legs drawn under the body. When an effort was made to have it "stand over," the horse moved with great difficulty, took short steps and flexed the joints as little as possible. The colicky symptoms disappeared in the course of a few days and the general condition of the animal improved. When the horse walked the legs were strongly flexed at the hocks, drawn forward with a jerk, held in the air for an instant and then dropped heavily to the ground. A trembling of the hind quarter accompanied this movement. In the weeks which followed, these symptoms extended to the fore limbs, so that in walking all of the joints would be strongly flexed. At this stage the hind legs would not be brought forward in a straight line, but followed a zig zag course. When the horse's head was covered it could be made to move only by great urging and its progress was very unstable.

Continued exercise helped the animal a little, but as it was subject to frequent relapses the owner sold it.

Magnin thinks that this was a case of locomotor ataxia, due to a lesion of the spinal cord, but the symptoms were not so characteristically developed as in man. (*Berl. Thierärz. Woch*, *vii, 32.*

---

### Iodoformed Ether to Prevent Loss of Skin after Firing.

By Prof. Nocard.

---

[*Recueil de Méd. Vét.*, *August, 1891.*]

To prevent the loss of skin, which is one of the gravest accidents that may follow firing, Professor Nocard recommends a spray of iodoformed ether. It causes the pain to disappear almost immediately in most cases, and when the eschar separates, the wound is found to be already covered by epidermis and in a fair way to cicatrization. Its good effects appear due to its power of suppressing pruritis and opposing bacterial infection.

